# Where are the horses? With the sheep or cows? Uncertain host location, vector-feeding preferences and the risk of African horse sickness transmission in Great Britain

**DOI:** 10.1098/rsif.2013.0194

**Published:** 2013-06-06

**Authors:** Giovanni Lo Iacono, Charlotte A. Robin, J. Richard Newton, Simon Gubbins, James L. N. Wood

**Affiliations:** 1 Disease Dynamics Unit, Department of Veterinary Medicine, University of Cambridge, Cambridge CB3 0ES, UK; 2 Animal Health Trust, Centre for Preventive Medicine, Newmarket CB8 7UU, UK; 3 The Pirbright Institute, Pirbright, Woking GU24 0NF, UK

**Keywords:** epidemiology, dilution effect, vector-borne disease, basic reproduction number, *Culicoides*

## Abstract

Understanding the influence of non-susceptible hosts on vector-borne disease transmission is an important epidemiological problem. However, investigation of its impact can be complicated by uncertainty in the location of the hosts. Estimating the risk of transmission of African horse sickness (AHS) in Great Britain (GB), a virus transmitted by *Culicoides* biting midges, provides an insightful example because: (i) the patterns of risk are expected to be influenced by the presence of non-susceptible vertebrate hosts (cattle and sheep) and (ii) incomplete information on the spatial distribution of horses is available because the GB National Equine Database records owner, rather than horse, locations. Here, we combine land-use data with available horse owner distributions and, using a Bayesian approach, infer a realistic distribution for the location of horses. We estimate the risk of an outbreak of AHS in GB, using the basic reproduction number (*R*_0_), and demonstrate that mapping owner addresses as a proxy for horse location significantly underestimates the risk. We clarify the role of non-susceptible vertebrate hosts by showing that the risk of disease in the presence of many hosts (susceptible and non-susceptible) can be ultimately reduced to two fundamental factors: first, the abundance of vectors and how this depends on host density, and, second, the differential feeding preference of vectors among animal species.

## 1. Introduction

A large body of ecological and epidemiological studies has highlighted the profound effects of spatial distributions of living organisms on population and disease dynamics (see [1], and references therein). This issue has also raised considerable interest outside the scientific community; inaccurate knowledge of spatial host distribution is regarded as a central problem for health authorities, especially in the presence of a sudden outbreak of disease when control measures need to be quickly implemented. As such information is often only partially available, developing mathematical tools that overcome the limited predictive capacity due to uncertainty in host distribution is a key scientific goal [2–4]. To this end, one study examined the specific case of foot-and-mouth disease spreading between farms where spatial clustering is ignored [1]. Tildesley *et al.* [1] showed that, if their model is carefully parametrized to match epidemic behaviour, then assuming that farms are randomly located within a region is sufficient to determine optimal control measures. The approach relies on an artificial parametrization that incorporates the complex effects of the spatial structure in the data [1] and was relevant for a disease where spatial ecological variability is not directly important; despite this, the approach is particularly appealing in the absence of precise demographic data. However, there are important examples where additional information, which can be used as a proxy for host locations, is available. Human travelling statistics have been assessed by analysing the circulation of bank notes in the USA [5]; spatio-temporal changes in population density have been measured by quantifying anthropogenic light from satellite imagery [6]; mobile phone technology has been used to collect data on social networks and behavioural data to explore the networks of transmission [7]. The spatial distribution of horses in Great Britain (GB) is another intriguing example, as the National Equine Database (NED) recorded only the address of the owners and not the actual location of the horses. Mapping owner addresses as a simple proxy for horse location is tempting but previous studies have shown that such an approach introduces an important source of error resulting in a spatial distribution biased towards large urban areas [8]. Errors in an unrealistic equine map could be further amplified in a disease risk map in the presence of strong environmental dependencies. This is the case, for instance, when the epidemiological parameters depend on the temperature, and therefore location, or when the presence of non-susceptible hosts for disease influences transmission.

Estimating the risk of African horse sickness (AHS) in GB is an illuminating case that exemplifies these issues, also being one of the most important equine diseases. AHS is a highly fatal, viral disease (the mortality can be as high as 90%) caused by the African horse sickness virus (AHSV), which is closely related to bluetongue virus (BTV). Similar to BTV, AHSV is transmitted and amplified by *Culicoides* biting midges. The outbreaks of bluetongue in 2006 [9,10] and the recent incursion of Schmallenberg virus [11,12] for ruminants are clear examples of incursions of novel species of arbovirus into Europe with important economic consequences [13–15]. Thus, the incursion and spread of BTV-8 in northern Europe has greatly increased the concerns of the GB equine industry, since AHSV could similarly be introduced, with potentially devastating economic and welfare consequences for the equine population and associated industries.

The risk of AHS spread depends on a range of both epidemiological and entomological factors, many with strong environmental dependencies. These include the temperature dependence of key virological parameters, the ecology of *Culicoides* species and how their abundance depends on host densities and the influence of non-susceptible hosts (e.g. ruminants, whose location in GB is known) kept in proximity to equine hosts; all these factors are spatially dependent. Thus, reliable data for the spatial distribution of horses in GB are of fundamental importance to assess the risk of the disease.

The role of non-susceptible hosts in mitigating or amplifying disease is poorly understood, although the idea of deploying a preferred host to protect man or animals from insect-borne disease has been suggested before [16]. However, the influence of non-susceptible hosts is complicated by two potential, but contrasting effects: a dilution effect, whereby *Culicoides* exhibit a feeding preference for a non-susceptible, non-equid host; and an amplification effect, whereby increased vertebrate host densities result in increased vector abundance. It is essential to disentangle these processes when assessing the risk of a disease.

Here, we address these issues by developing a credible distribution of horses in GB that can be used to re-assess, in the light of current knowledge, the risk of AHSV spread in GB and the efficacy of potential control measures. To this end, we combined NED and land-use data in a Bayesian framework, developing an algorithm to infer a realistic distribution for the location of horses. Using this inferred distribution of horses, we estimated the spatial and temporal variation in risk by computing the basic reproductive number *R*_0_ (the average number of secondary cases arising from the introduction of a single infected individual to an otherwise susceptible population) [17,18]. In particular, we explore the impact of non-susceptible vertebrate hosts of the risk of transmission and the efficacy of vaccination.

## 2. Material and methods

### 2.1. Developing a credible national distribution of horses in Great Britain

Previous studies [8,19,20] have shown that the distribution of NED-registered horse owners does not mirror the distribution of locations where the corresponding horses are kept. In particular, a survey of NED-registered owners (1009 samples) provided complete postcode records for owners and their corresponding horse locations [19], and revealed an inverse relationship between built-up land use and the proportion of horses kept at the same postcode as owners' addresses [8]. Data from the same survey also showed that the distribution of the horse-owner distances was well described by a power-law distribution, irrespective of the local values of built-up coverage (see §3.1).

Heavy-tailed distributions are compatible with the reasonable assumption that suitable horse premises in the neighbourhood of the owner's address are the most preferred locations, although cases of large owner–horse separations are not precluded. Based on this information, we combined available NED and land-use data [21] in a Bayesian approach to develop a plausible national distribution of horses in GB.

#### 2.1.1. Mechanistic model

A mechanistic model was formulated that provided the conditional probability 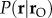 of a horse being kept at position **r** = (*x*,*y*) when we know the owner's location 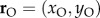. The modelling approach combines: (i) the empirical, inverse relationship between built-up land use and the proportion of horses kept at the same postcode as the owners' addresses [8]; and (ii) a fat-tailed spatial kernel allowing a non-negligible probability of large owner–horse separations. Accordingly, the probability 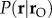 that a horse is kept at the location **r** = (*x*,*y*) when we know the owner's location 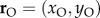 can be written as2.1
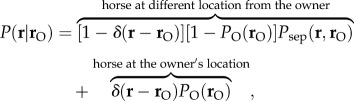
where *δ* is Dirac delta function. The probability *P*_O_(**r**) reflects the suitability of a location for keeping horses (e.g. rural area with the presence of stables) and is related solely to land use. In particular, *P*_O_(**r**_O_ ) can be interpreted as the probability that a horse is kept at the owner's location 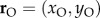. If the horse is not kept at the same location as the owner, 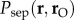 gives the probability that the horse is located at position **r** = (*x*,*y*) at a Euclidean distance 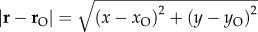 from the owner's location. This is modelled as the joint probability2.2
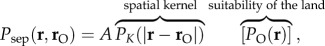
where 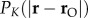 is the probability that the horse and owner locations are a given distance, 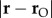, apart. The constant *A* is fixed by the constraint: 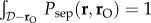, where *𝒟* − **r**_O_ represents the entire spatial domain (i.e. GB except the owner's location). This constraint ensures that each horse is associated with one and only one location (although it is possible that more than one horse may be kept at the same location, as occurs in stables).

Complete postcode records for 1009 owners and their horses were available from a survey of NED-registered owners [19]. These were used to estimate the distribution of the distances between the two. A visual inspection of this empirical distribution, which is related to *P*_sep_, revealed a large scatter of the data. This suggests that, although 92 per cent of horses resided within 10 km of their owners [19], there is a non-negligible probability of large horse–owner separations comparable to length scales at country level (e.g. the owner might reside in southern England and the horse in Scotland). This behaviour can be captured by modelling the spatial kernel *P*_K_ as a fat-tailed distribution,2.3
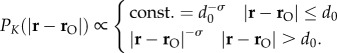
Underlying this choice is the expectation that suitable horse locations in the neighbourhood of the owner's address are favoured.

Data on built-up coverage (the fraction of built-up surface) [21] were obtained for each of the 1009 postcode records for owners and their horses from the NED-registered owner survey. The probability, *P*_O_, that a horse is kept at the owner's location, **r**_O_, given the local value of built-up coverage *u*(**r**_O_) was modelled as2.4
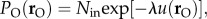
where *N*_in_ is a normalization constant to ensure 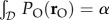 when integrated over the entire domain (i.e. GB). The parameter *α* represents the fraction of horses kept at the owner′s location (according to the empirical NED-registered owner survey, *α* = 0.7).

#### 2.1.2. Parameter estimation

Parameters in the model were estimated using Bayesian inference. In this case, the likelihood for the model parameters (*d*_0_, *σ*) is2.5
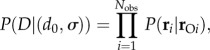
where *D* represents the set of observed data, *N*_obs_ is the number of records from the NED-registered owner survey, and 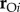 and **r**_*i*_ are the positions of the owner and horse, respectively, for the *i*th record. We assumed non-informative prior distributions for all parameters.

The parameters *d*_0_ and *σ* were estimated by using a Markov chain Monte Carlo (MCMC) approach to generate samples from the joint posterior distribution for the parameters (figure SB-1, electronic supplementary material). However, the parameter *λ* was fixed at its least-squares estimate (*λ* = 8.76) to reduce computational cost (figure SB-2, electronic supplementary material). One chain of 95 000 iterations was run using the MCMCpack package in R [22], with the first 5000 iterations discarded to allow for burn-in of the chain. Convergence of the chain was monitored visually and using standard diagnostics. Posterior estimates (mean and 95% credible interval (CI)) for the parameters are *d*_0_ = 1071 (95% CI 208–2772) and *σ* = 2.82 (95% CI 2.37–3.45).

#### 2.1.3. A map of the locations of horses in Great Britain

The NED provides the number of owners present at 8670 locations (here we identify each location by the index *i* and the associated number of owners by 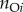). There were up to 10 667 owners at any one location, with the highest number of owners occurring in the Newmarket postcode sector, resulting in a density of a few hundred horses per square kilometre. For each owner location, 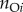 corresponding horse locations were generated according to the conditional probability 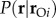 in equation (2.1) using the Metropolis algorithm. The simulation was implemented by using the R package MCMC [23], sampling (at least) every 1000 iterations. Considering the large number of simulations required (each of the 8670 different locations requires an independent simulation) diagnostic analysis was done for a randomly selected sample of simulations.

### 2.2. Basic reproductive number *R*_0_

The transmission model underlying the current host–vector model is similar to that described previously for BTV [18,24]. The basic reproductive number *R*_0_ was calculated by using the next-generation matrix (NGM) approach [17]. For AHS, the NGM has elements, *k*_*hl*_, given by the expected number of infections of type *h* (either horse (H) or vector (V)) arising from a single infected individual of type *l*, so that2.6
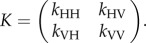
Two elements of the NGM are straightforward to derive, because there is little or no direct transmission between horses (i.e. *k*_HH_ = 0) or between vectors (i.e. *k*_VV_ = 0). The two elements representing transmission from vector to horse (*k*_HV_) or horse to vector (*k*_VH_) are computed as follows.

*Transmission from vector to horse.* Once a vector takes an infected blood meal, it must complete the extrinsic incubation period (EIP), i.e. latent period, before it becomes infectious. The EIP is assumed to follow a gamma distribution with mean 1/*ν* and variance 1/(*n*_V_*ν*^2^) [25], where *n*_V_ is the scale parameter (table 1). If the vector mortality rate is *μ*, the probability that a vector survives the EIP (and so becomes infectious) is 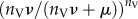. Following completion of the EIP the vector will survive for 1/*μ* days, during which time it will bite susceptible horses *aϕ* times per day (here *a* is the reciprocal of the time interval between blood meals and *ϕ* is the proportion of bites on horses), and a proportion, *b*, of these bites will result in an infected horse. Consequently, the expected number of infected horses arising from a single infected vector is given by2.7
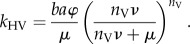

**Table 1.** Epidemiological parameters for the transmission of African horse sickness virus (AHSV). BTV, bluetongue virus.

description		symbol	estimate or range	comments
probability of transmission from vector to host		b	beta (7.38,2.12)	based on an analysis of data on the transmission of BTV to sheep presented in [26] (cf. [27]); full details are presented in the electronic supplementary material
probability of transmission from host to vector		*β*	beta (1.05,39.6)	based on experimental infection of field-caught *Culicoides imicola* with a number of different strains of AHSV [28]; full details are presented in the electronic supplementary material
reciprocal of the time interval between blood meals		a	—	depends on temperature, *T*, such that: *a* = 0.0002*T* (*T* − 3.7)(41.9 − *T*)^1/2.7^ [29]
duration of viraemia	mean	1/r_H_	uniform (4,8)	assumed to follow a gamma distribution with mean (1/*r*_H_) and variance (1/*n*_H_*r*_H_^2^ ); estimates for the mean were obtained from [30]
	scale parameter	n_H_	uniform (7,15)	
disease-associated mortality rate		d_H_	uniform (0.28,0.52)	based on a case fatality of 75–90% [30]; full details are presented in the electronic supplementary material
maximum vector-to-host ratio	only horses	m	uniform (0,10 000)	based on a maximum host biting rate (*ma*) of 2500 bites per host per day [31], and light trap catches of up to 10 000 individuals per night [32]; the vector-to-host ratio varies seasonally as *mδ*(*t*), where *δ*(*t*) is the seasonal vector activity (see below). In general, the ratio is rescaled by a factor *f* depending on the density of other non-susceptible hosts. Other regimens (table 2) are also considered
	horses and non-susceptible hosts	m	uniform (0,10 000)*f* 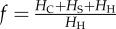	
extrinsic incubation period (EIP)	mean	1/*ν*	—	follows a gamma distribution with mean (1/*ν*) and variance (1/*n*_*ν*_ *ν* ^2^ ); the mean EIP depends on temperature, *T*, so that: 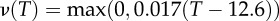 [25]
	scale parameter	*n*_*ν*_	17	
vector mortality rate		*μ*	—	depends on temperature: *μ* = 0.009exp(0.16*T*) [33]
seasonal vector activity		*δ*	—	given by 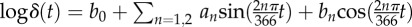 ; note that this function was normalized, so that the maximum value is equal to 1; parameters are: *b*_0_ =−1.71, *a*_1_ =−1.56, *a*_2_ =−1.49, *b*_1_ =−3.74, *b*_2_ =−1.00 [34]

**Table 2.** Expression for the basic reproductive number *R*_0_^NSH^ and the conditions to reduce *R*_0_^NSH^ below 1 owing to the introduction of non-susceptible hosts.

regimen I: number of *Culicoides* midges is not altered by the introduction of non-susceptible hosts	
number of vectors	*N*_Vectors_ unchanged
basic reproductive number in the absence of non-susceptible hosts	*R*_0_ from equation (2.10); *ϕ* = 1, *m* = *N*_Vectors_/*H*_H_
basic reproductive number in the presence of non-susceptible hosts	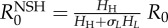
condition 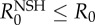	always satisfied
condition 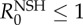	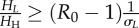
regimen II: number of *Culicoides* midges linearly increases with the number of horses and other livestock	
number of vectors	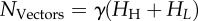
basic reproductive number in the absence of non-susceptible hosts	*R*_0_ from equation (2.10); *ϕ* = 1, *m* = *γH_H_*/*H*_H_ = *γ*
basic reproductive number in the presence of non-susceptible hosts	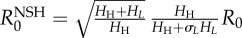
condition 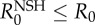	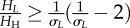
condition 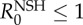	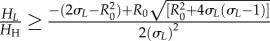
regimen III: number of *Culicoides* midges increases as the power of *ρ* with the number of horses and other livestock	
number of vectors	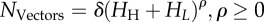
basic reproductive number in the absence of non-susceptible hosts	*R*_0_ from equation (2.10); *ϕ* = 1, *m* = *δH*_H_^*ρ* −1^
basic reproductive number in the presence of non-susceptible hosts	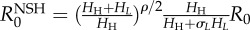
condition 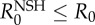	the condition is always satisfied for *σ*_*L*_ ≥ 1 and *ρ* ≤ 2. In general, the condition depends on a threshold *r*_*th*_ that decreases monotonically with *σ*_*L*_ and has to be computed numerically. For *ρ* > 2, then 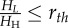 . For *ρ* < 2 then 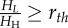
condition 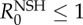	in general, the condition depends on thresholds *r*_*th*_ that have to be computed numerically
regimen IV: number of *Culicoides* midges scales arbitrarily with the number of horses and other livestock	
number of vectors	*N*_Vectors_ = *f*(*H*_H_ + *H*_*L*_ ), *f* is an arbitrary function
basic reproductive number in the absence of non-susceptible hosts	*R*_0_ from equation (2.10); *ϕ* = 1, *m* = *f*(*H*_H_ )/*H*_H_
basic reproductive number in the presence of non-susceptible hosts	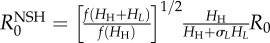

*Transmission from host to vector*. The duration of viraemia in horses (assumed to indicate infectiousness) was assumed to follow a gamma distribution with mean 1/*r*_H_ and variance 1/(*n*_H_*r*_H_^2^), where *n*_H_ is the scale parameter (table 1). If disease-associated mortality occurs at a rate *d*_H_, the mean duration of infectiousness is given by 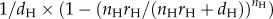. During this time period, a host is bitten by susceptible midges on average *maϕ* times per day (here *m* is the vector-to-host ratio), a proportion, *β*, of which become infected. Hence, the expected number of infected vectors arising from a single infected horse is given by2.8
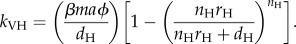
Some linear algebra shows that the dominant eigenvalue of the NGM (i.e. *R*_0_) is2.9
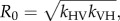
which, on substituting the expressions for *k*_HV_ and *k*_VH_, yields2.10
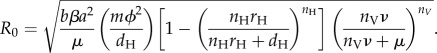
The vector-to-host ratio and the proportion of bites were calculated as2.11
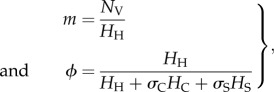
where *H*_H_, *H*_C_ and *H*_S_ are the population of horses, cattle and sheep; *N*_V_ is the number of vectors representing the abundance of *Culicoides*, *σ*_C_ and *σ*_S_ are measures of vector preference for cattle and sheep compared with horses: if 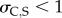, vectors feed preferentially on horses, otherwise they feed preferentially on cattle/sheep. In the non-spatial analysis, we assumed that only one type of non-susceptible host was present. In this case, the proportion of bites reduces to: 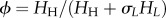, where *H_L_* is the population of non-susceptible hosts and *σ_L_* the corresponding vector preference. The risk of AHS is potentially influenced by the presence of other non-susceptible animals, such as goats and wild ruminants. However, the impact of these animals is expected to be negligible in GB because of their limited abundance (approx. 88 000 and 500 000–600 000, respectively, while the number of cattle and sheep is 10 and 36 million, respectively; see www.archive.defra.gov.uk).

Plausible ranges rather than point estimates were considered for most epidemiological and virological parameters, which constitute *R*_0_ (table 1). Where possible, estimates applicable to GB and AHS were used; otherwise, data for other species and countries were used (for details, see table 1). Replicated Latin hypercube sampling (LHS) was used to explore the parameters influencing the basic reproduction number, *R*_0_ (see [18] and references therein). The LHS results were used to compute the mean and maximum values for *R*_0_. Results are based on 500 replicates in non-spatial cases and 100 replicates in the spatial cases.

### 2.3. Seasonal maps for *R*_0_

The spatial distribution of *R*_0_ was calculated at the same level of resolution as the NED owners' data, i.e. postcode sectors, an abbreviated form of address (e.g. CB8 9) used in GB. There are approximately 9000 postcode sectors in GB, each containing approximately 3000 addresses (see www.ons.gov.uk). The size of postcode sectors varies, ranging from 2864 km^2^ in a low-populated region in the Scottish Highlands to approximately 0.001 km^2^ in most of the densely populated sectors of London.

Temperature data for 2006 were used, because this was an exceptionally warm year, with all GB regions recording their warmest rolling 12-month period. Monthly averaged mean temperatures were obtained from the BADC/MIDAS database (see http://badc.nerc.ac.uk/view/badc.nerc.ac.uk__ATOM__dataent_ukmo-midas). Seasonality in vector activity was obtained from an analysis of data from a network of 12 suction traps in England, covering a variety of habitat types [34] (table 1).

## 3. Results

### 3.1. A credible national distribution of horses in Great Britain and its impact on risk predictions

**Figure 1.**
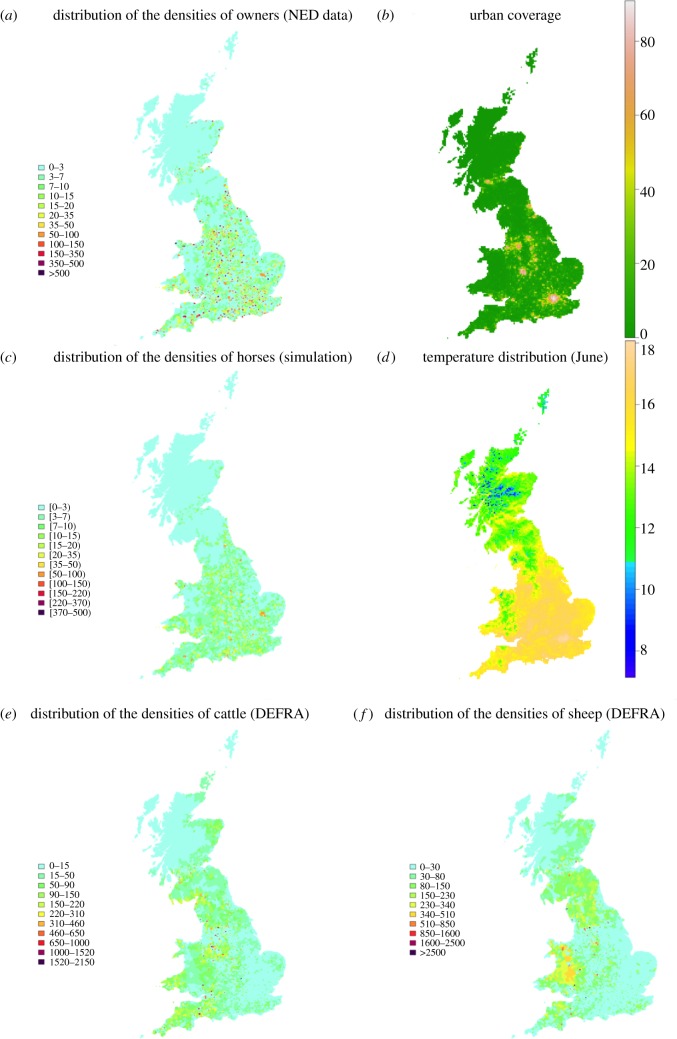
Spatial distribution of horse-owners and horses. (*a*) Number of horse-owners divided by the area of the postcode sector where they are located; NED data. (*b*) Percentage of built-up coverage [21]. Horses are expected to be located in rural areas. (*c*) Number of horses divided by the area of the postcode sector where they are located; data from simulation according to the correction algorithm. (*d*) Spatial distribution of temperature, June 2006. (*e*) Spatial distribution of densities of cattle per postcode sector (see www.archive.defra.gov.uk). (*f*) Spatial distribution of densities of sheep per postcode sector (see www.archive.defra.gov.uk). DEFRA, Department for Environment, Food and Rural Affairs.

**Figure 2.**
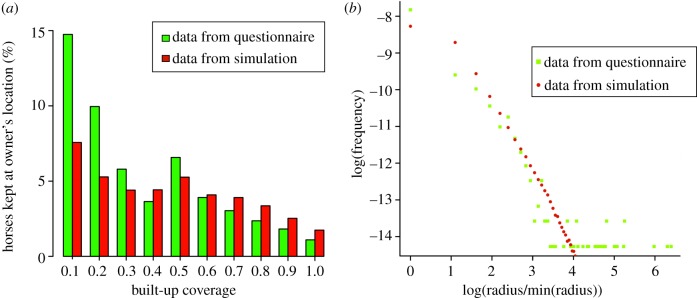
Data on factors influencing horse and owner locations. (*a*) Relationship between the proportion of horses kept at the owner's postcode and local built-up coverage. (*b*) Observed and simulated distribution of horse–owner separations (note the logarithmic scale). The separation is calculated as the Euclidean distance between the owner's location and the simulated position of the horse, irrespective of the built-up coverage. The observed data are from a sample (1009 records) of NED-registered owners [19]. (Online version in colour.)

The distribution of owners (figure 1*a*) strongly mirrors urban coverage (figure 1*b*); in particular, in two highly urbanized locations (City of Westminster and one of the Greater London boroughs) where the density of owners is exceptionally high (more than 2000 owners per square kilometre), although the actual horse density is low in these locations. Re-distribution of the NED data according to the algorithm developed here appears to correct this source of bias (figure 1*c*) with the re-distributed horse population more evenly spread out towards rural areas and the exceptionally high densities in urban settlements being removed. The output of the correction algorithm was compared with the sample from the NED survey, which showed that it is governed by the same statistics (figure 2).

**Figure 3.**
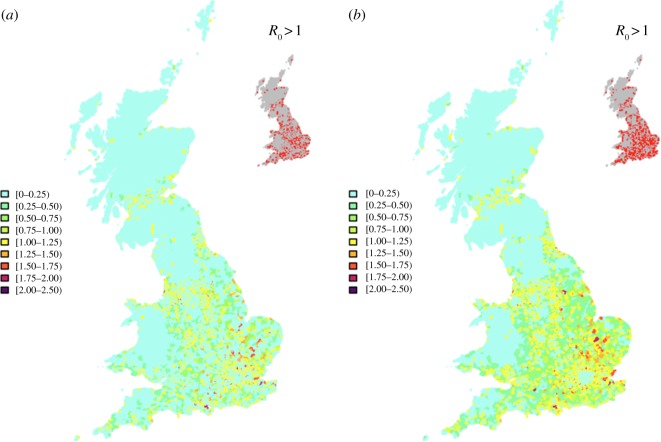
Impact of the correction algorithm on the predictions for *R*_0_. Spatial distribution of *R*_0_ based on: (*a*) owner addresses as a proxy for horse locations; (*b*) redistributed horse locations based on the correction algorithm. Areas where *R*_0_ > 1 are also shown in the inset. No other non-susceptible hosts are included.

The highly clustered distribution of owners results in a sparse distribution of *R*_0_ with many postcode sectors having values less than 1 (figure 3*a*). A more realistic distribution of horses, however, leads to a more even distribution and, most importantly, more locations where *R*_0_ > 1 as shown in the insets (figure 3*b*).

### 3.2. Temporal and spatial variations of the risk of an outbreak of African horse sickness in Great Britain

**Figure 4.**
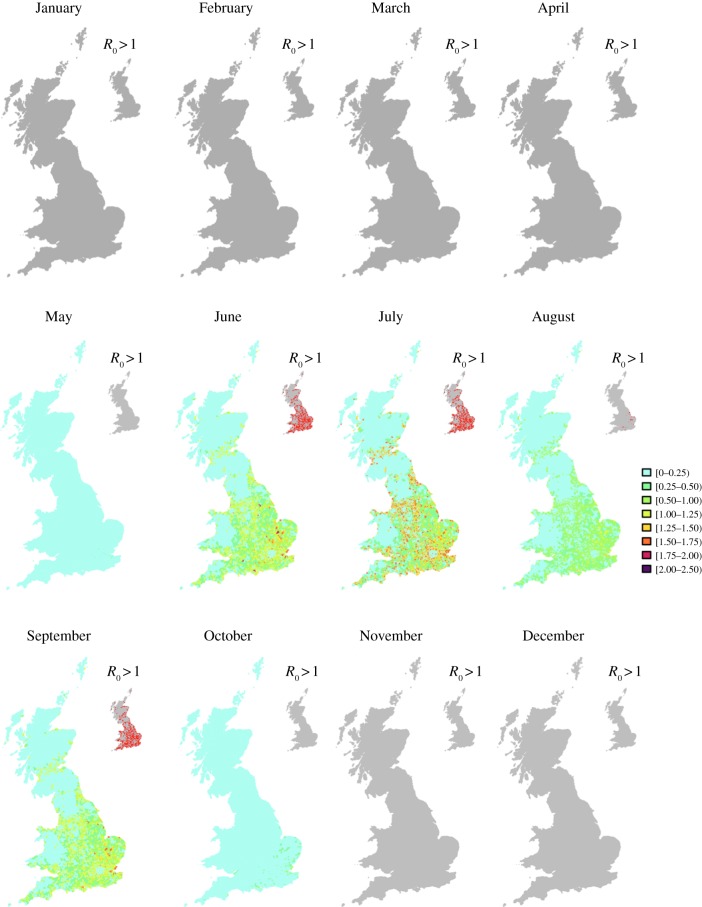
Spatial distribution *R*_0_ for different months. The risk of an epidemic is negligible (*R*_0_ = 0 on average) in cold months (January–April; November–December) and reaches its peak in July. The patterns are largely influenced by the distribution of horses, the presence of other non-susceptible hosts (cattle and sheep) and the abundance of *Culicoides*. Here, we assume that *Culicoides* have no preference towards any particular host, and their abundance is proportional to the host density. Areas where *R*_0_ > 1 are also shown in the inset.

Figure 4 shows spatial variation of mean *R*_0_ and the locations where *R*_0_ > 1 from January to December based on temperature data for 2006 (maximum values are shown in figure SC-1, electronic supplementary material). It is evident that the risk of AHS reflects the spatial distribution of temperature (figure 1*d*), where, for instance, higher values for *R*_0_ occur in warmer regions such as southeast England. It is also driven by the seasonally varying activity of *Culicoides* (which is lower in August than in July and September, see table 1; [34]). For instance, *R*_0_ in August is lower than that in September despite the average mean temperature being similar (15.3°C in August and 15.6°C September). Furthermore, the distribution of horses influences the magnitude of *R*_0_. For example, the lowest values of *R*_0_ occur in the London area, where the number of horses is small and the number of livestock negligible (figure 1*e*,*f*) despite the high temperatures.

#### 3.2.1. Influence of non-susceptible hosts and effect of vector abundance on *R*_0_

**Figure 5.**
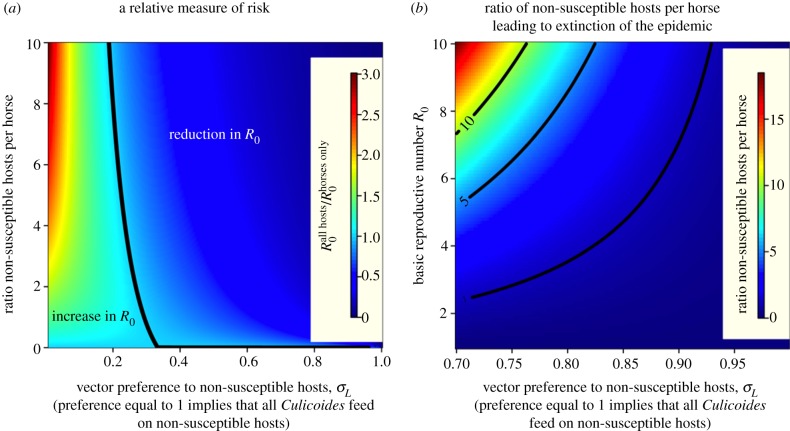
Influence of non-susceptible hosts on *R*_0_. (*a*) Ratio of *R*_0_ estimated in the presence of all hosts relative to *R*_0_ estimated in the presence of horses only as a function of the vector preference *σ*_*L*_ and non-susceptible host to horse ratio. When this relative value is less than 1 then it is advantageous to introduce non-susceptible hosts. (*b*) The ratio of non-susceptible hosts relative to the number of horses expected to lead to *R*_0_ < 1. The abundance of *Culicoides* midges is proportional to the abundance of the local hosts.

The influence of non-susceptible hosts on the basic reproductive number, denoted here as *R*_0_^NSH^, is essentially driven by the feeding preference *σ*_*L*_, the vector abundance *N*_V_ and its dependency on host population size. In table 2, we present the analytical expression for *R*_0_^NSH^ and the conditions leading to *R*_0_^NSH^ < 1 for different scenarios. The simplest scenario (regimen I) corresponds to the case when the population of *Culicoides* midges is not altered by the introduction of an alternative vertebrate host. Underlying this choice is the assumption that the key factor in the ecology of *Culicoides* midges is land use. This assumption is likely to be unrealistic as one would expect that the abundance (owing to survival and active search) of *Culicoides* increases with the resource available (e.g. linearly in regimen II and as a power law in regimen III). Regimen IV represents the more general case when the population of *Culicoides* depends on an arbitrary function of the total host population. As an illustrative case, we considered a scenario in regimen II with *H*_H_ equids and *H_L_* non-susceptible hosts and calculated the basic reproductive number in the presence of all hosts relative to that in the presence of horses only, i.e. the ratio of 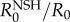. When this ratio is below 1, then it is advantageous to keep non-susceptible hosts in the proximity of horses. As shown in figure 5*a*, two distinct regions (
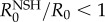
 and 
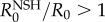
) can be identified. The extension of each region is delimited by the vector preference *σ*_*L*_ and the ratio of non-susceptible hosts to horses (*H_L_*/*H*_H_). The existence and shape of such regions depends on the particular regimen for vector abundance. If the condition 
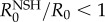
 is satisfied then the ratio of non-susceptible hosts per horse leading to the extinction of the disease can be estimated (figure 5*b*). These conditions depend solely on the basic reproductive number *R*_0_ in the absence of alternative hosts, the number of equids, the number of non-susceptible hosts and the vector preference *σ*_*L*_. The effect of vector abundance on the basic reproductive number, in the presence of horses only, can be readily investigated from the analytical solution displayed in table 2 with the condition *H_L_* = 0.

**Figure 6.**
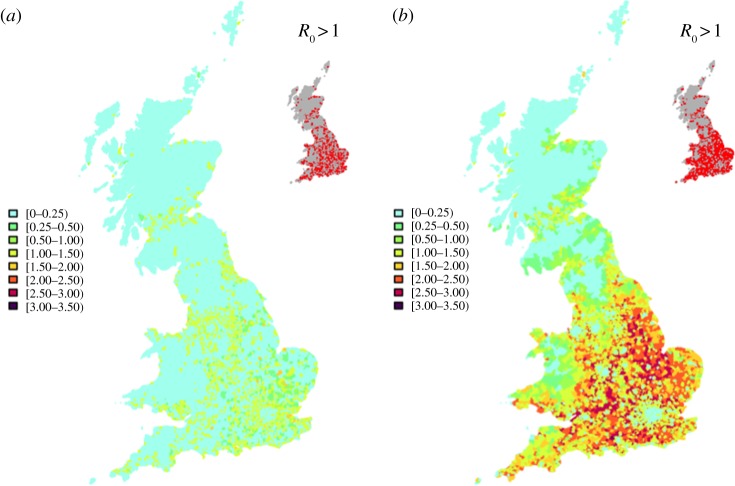
Effect of feeding preference on the spatial distribution of *R*_0_. (*a*) Distribution of *R*_0_ when *Culicoides* midges exhibit a large feeding preference (eightfold) towards cattle. The distribution of cattle is also shown in figure 1*d*. (*b*) Distribution of *R*_0_ when *Culicoides* midges exhibit large feeding preference (eightfold) towards horses rather than cattle and sheep. Temperature based on June 2006.

The influence of alternative hosts in the spatial case is highlighted by the map of *R*_0_^NSH^ in July for two contrasting values of the vector feeding preference: high preference towards cattle and sheep and high preference towards horses (figure 6).

#### 3.2.2. Impact of vaccination

**Figure 7.**
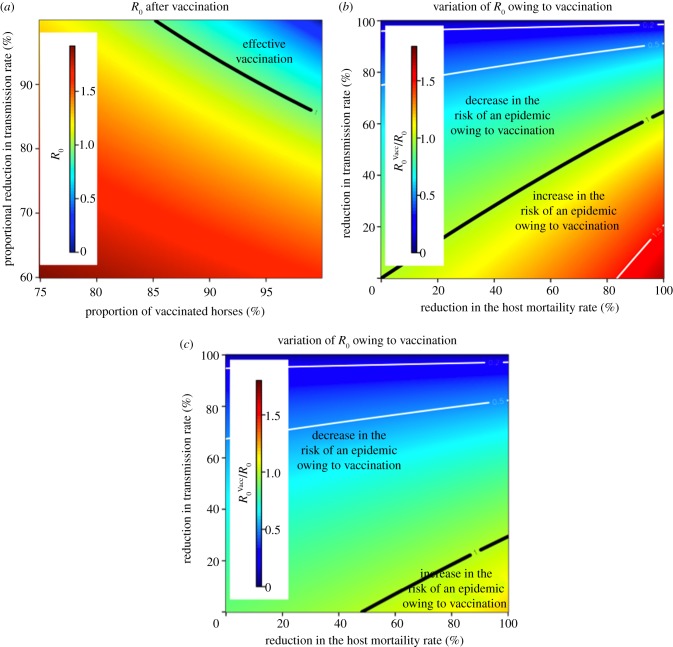
Effect of vaccination on *R*_0_. (*a*) *R*_0_ owing to vaccination as a function of the proportion of vaccinated horses and the reduction factor in the transmission rate. Parameters in the top-right region are expected to lead to the extinction of the epidemic. The initial value of *R*_0_ for un-vaccinated horses is 2.6, which leads to a vaccination coverage (for a uniformly mixing host–vector population) greater than 100(1−1/2.6^2^) = 85% in order to block transmission. (*b*) Relative variation of *R*_0_ when 100% of horses are vaccinated compared with *R*_0_ in the absence of vaccinated horses as a function of the reduction in the host mortality rate and the reduction factor in the transmission rate. A sensible reduction in the host mortality rate might lead to an increase in *R*_0_. (*c*) As in (*b*) but the mean duration of viraemia is assumed to be reduced by 50% as an additional effect of vaccination. Temperature 22.5°C, *b* = 0.7530, *β* = 2.7 × 10^−2^, *d*_H_ = 0.4, 1/*r*_H_ = 6, 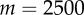, *n*_H_ = 4, unbiased feeding preference.

Vaccination is a principal control measure for AHS [30]. For a perfect vaccine which renders the host immune to infection, the fraction of vaccinated horses required to reduce 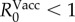 (vaccination coverage) depends on the basic reproductive number as 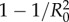, where 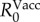 and *R*_0_ are the basic reproductive numbers is the presence and the absence of vaccinated horses, respectively [35]. For an imperfect vaccine, for instance one that reduces the transmission rate or the mean duration of viraemia, this ideal vaccination coverage must be rescaled by an appropriate reduction factor depending on the parameters affected by the vaccine (see the electronic supplementary material). However, this critical vaccination coverage ought to be seen as an upper limit, as it is based on the assumption that the host and vector populations mix uniformly. Figure 7*a* shows the value of 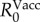 as a function of the proportion of vaccinated horses and the reduction factor in the transmission rate. The top-right region in parameter space is characterized by 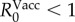 and therefore extinction of the epidemic. Here, the effect of vaccination is assumed to reduce the probability of transmission (either from host to vector *b* or from vector to host *β*) and the mean duration of viraemia by 50 per cent.

Vaccination is likely to affect other virological parameters, including a reduction in the rate of mortality of the host. As a host will live longer, this might increase the risk of an epidemic if not compensated by a reduction, for example, in the transmission rate and/or the mean duration of viraemia. To this end, we considered a thought experiment in which vaccination reduces only the transmission rate and the mortality rate. This is illustrated in figure 7*b*, which displays the ratio of the basic reproductive number *R*_0_^Vacc^ (for 100% of the vaccinated horses) when compared with *R*_0_ in the absence of vaccinated horses. For a more realistic case, when vaccination is also assumed to reduce the mean duration of viraemia, the effect could persist but the region of parameter space leading to an increase in *R*_0_ is smaller (figure 7*c*).

## 4. Discussion

The current work has addressed a number of issues in spatially explicit epidemic modelling of vector-borne disease, exemplified by the important case of AHS, relating to host location, the evaluation of dilution effects when vectors may feed on both susceptible and non-susceptible hosts (a key factor that is frequently ignored) and the effect of vaccination.

The work highlights the importance of a credible host distribution when assessing the risk of a disease and the impact of control. Previous studies have shown that the distribution of NED-registered horse owners does not mirror the distribution of locations where the corresponding registered horses are kept [8,19,20]. We showed that mapping owner addresses as a simple proxy for horse location underestimates the risk of an outbreak of AHS in GB. To prevent this problem, a correction algorithm was implemented to infer a more realistic distribution of the equine population in GB. The correction algorithm was built over the empirical dependence between spatial separation and land use combined with NED data in a Bayesian framework. Inferring spatial knowledge from related information used as a proxy is an increasingly common approach [5–7]. The approach formulated in the present study provides an additional tool for this class of problems.

Combining the new host spatial distribution with existing national data on ambient temperatures at different times of the year, seasonal abundance of *Culicoides* and the distribution of other host species (especially cattle and sheep) resulted in a meaningful spatio-temporal assessment of the risk of AHS in GB. The modelling framework was built on contributions by Lord *et al.* [36–38], Backer & Nodelijk [27] and Gubbins *et al.* [18]. An important finding, in agreement with [18], was that the risk of AHS is strongly affected by temperature, being higher in warmer regions or warmer years. This is a particular source of concern as climate change has also been associated with alterations of *Culicoides* distributions and consequently their associated diseases [39].

The risk is also driven by the seasonally varying abundance of *Culicoides*. Using sensitivity analysis, Lord *et al.* [37] identified *Culicoides* population size as one of the most important factors in determining whether or not an epidemic occurred and in influencing the size of the epidemic. A key problem is that measurements of vector abundance and distribution are affected by the methodologies used (e.g. UV light/suction trap, CO_2_ trap, animal-baited drop trap). Direct collection of *Culicoides* from animals is considered the most reliable method for measuring biting rate [40–42]. However, owing to a paucity of such data, determining vector abundance and vector-to-host ratios accurately remains challenging.

In the particular case of *Culicoides*, the vector-to-host ratio is known anecdotally to vary by several orders of magnitude according to a wide variety of factors. Also, most studies do not investigate how the abundance of *Culicoides* is affected by the densities of available hosts. For example, two putative vectors of BTV (*Culicoides dewulfii* and *Culicoides chiopterus*), and potentially AHSV, develop as larvae in cattle dung [43,44] and, hence, would only be expected to come into contact with horses through overlapping host populations. The hypothesis that *Culicoides* abundance is density dependent is supported by recent findings that *Culicoides* abundance was significantly higher at trap locations with a high density of cattle in the locality [34]. Another study suggested that catches in light traps increase linearly with sheep numbers, at least for small host numbers [45]. Although the particular design of this experiment (low number of sheep, the presence of only one host, no habitat variations, measurements based on light trap catches) prevents robust generalizations, the findings are compatible with the common assumption of a fixed vector-to-host ratio [18,37,38].

Previously, Lord *et al.* [36] calculated *R*_0_ under different hypothetical relationships between vector population dynamics and either host or vector density, though assuming no vector preference for different hosts. Here, we have shown that knowledge of *Culicoides* abundance alone is not sufficient to discriminate whether the presence of non-susceptible hosts is beneficial or not and information on the feeding preference is essential. Despite a growing body of research that has focused on feeding patterns of midges [16,46–49], reliable measurements suitable for use in epidemiological studies are still scarce. The probability of taking a blood meal on a particular animal depends not only on its attractiveness but also on the numeric availability of a host. A common limitation in these studies is that knowledge of host abundance is only approximate.

To the authors' knowledge, the joint effects of *Culicoides* abundance and feeding preferences have not been rigorously investigated. If robust, accurate measurements of these factors were available, the current framework could be readily used to assess whether or not the proximity of non-susceptible hosts is likely to reduce or increase the risk of an AHS epidemic. In the absence of reliable measurements, we explored different hypotheses on the abundance of *Culicoides*. A key focus of the current work was to explore *R*_0_ and its dependence on (i) vector abundance and its relationship with the density of susceptible and non-susceptible host species and (ii) vector-feeding preferences between hosts. This allowed us to provide quantitative estimates for this potential dilution effect.

A variety of vaccines have been developed to prevent AHSV infection (see [50], and references therein). These include inactivated and live attenuated virus vaccines, virus-like particles produced from recombinant baculoviruses, a recombinant vaccinia-vectored vaccine and a DNA vaccine. Polyvalent cell culture attenuated vaccines are still routinely used for protective immunization of horses in sub-Saharan Africa to achieve sufficient protection against all nine AHSV serotypes. However, the simultaneous administration of multiple vaccine strains can result in interference during vaccine virus replication, possibly resulting in incomplete immunity [51,52]. For example, a recent study has shown that immunized horses in an AHS endemic area were infected with AHSV over a 2 year period [53]. Our results suggest that incomplete immunity with a reduction in the mortality rate of the horses might lead to an increase in the basic reproduction number (figure 7). It is conceivable that in a more realistic case, e.g. when vaccination sensibly reduces the mean duration of viraemia, this risk could be negligible. However, this emphasizes the need for accurate measurements of all virological parameters for live attenuated vaccines. By contrast, inactivated vaccines, such as the recombinant canarypox virus-vectored vaccine described by Guthrie *et al.* [50], results in a suppression of viraemia and no risk of transmission, and these are promising vaccine candidates for use in non-endemic areas, such as Europe.

In the event of a vaccination campaign, a key epidemiological parameter is the proportion of vaccinated horses required to generate herd immunity. For a perfect vaccine, such vaccination coverage is given by 1 − 1/*R*_0_^2^ but for an imperfect vaccine this threshold must be rescaled using an appropriate reduction factor depending on the parameters affected by the vaccine. In general, this leads to predictions that the required level of vaccination coverage is high (in figure 7*a*, with *R*_0_ for unvaccinated horses equal to 2.6, the critical coverage is 85%). Such a high prediction for vaccine coverage is not surprising; for example, Lord *et al.* [38] estimated that the prevention of 50 per cent of epidemics required 75 per cent coverage of horses and donkeys or 90 per cent coverage of horses only.

In the current work, spatial clustering, e.g. horses kept in livery yards, was not incorporated in the model. At the resolution used, this is expected to have little impact since estimations of the range of the spatial movement of *Culicoides* [54] are comparable with the typical sizes of the postcode sectors; the choice also captures the expectation that movement of *Culicoides* is reduced in highly urbanized areas (i.e. smaller areas of the postcode sectors) as streets act as barriers to disease vectors [55].

In the present model, the movement of horses was not included, despite their potential impact on transmission. To the authors' knowledge, data on horse movements in GB are limited, and modelling horse movement between countries has been proved to be challenging owing to large uncertainty in model inputs [56]. More importantly, one of the first actions following confirmation of AHS in the UK would be a movement restriction zone [57] and possibly a national movement ban on all equids. These considerations led to the choice of focusing our analysis on a local measure of disease risk (*R*_0_ at a particular location and time).

In summary, we have shown how it is possible to address the problem of inaccurate spatial demographic data by exploiting the partial information available. Here, combining NED and land-use data in a Bayesian approach, we developed an algorithm to infer a realistic distribution for the location of horses. Based on such a credible distribution of the host, we explored the impact of using inaccurate maps of equine distribution in predicting risk. In addition, we have clarified the role of non-susceptible hosts by showing that the risk of disease in the presence of many hosts (susceptible and non-susceptible) can be ultimately reduced to two fundamental factors: (i) abundance of vectors and how this depends on host density and (ii) differential feeding preference among animal species. Our results here identify key measurements needed for a better understanding of the elusive role of non-susceptible hosts.
